# Microstructure and Sliding Wear Behaviour of In-Situ TiC-Reinforced Composite Surface Layers Fabricated on Ductile Cast Iron by Laser Alloying

**DOI:** 10.3390/ma11010075

**Published:** 2018-01-05

**Authors:** Damian Janicki

**Affiliations:** Welding Department, Faculty of Mechanical Engineering, Silesian University of Technology, Konarskiego 18A, 44-100 Gliwice, Poland; damian.janicki@polsl.pl; Tel: +48-32-2371649

**Keywords:** in-situ composite, TiC, laser surface alloying, diode laser, ductile cast iron, sliding wear

## Abstract

TiC-reinforced composite surface layers (TRLs) on a ductile cast iron EN-GJS-700-2 grade (DCI) substrate were synthesized using a diode laser surface alloying with a direct injection of titanium powder into the molten pool. The experimental results were compared with thermodynamic calculations. The TRLs having a uniform distribution of the TiC particles and their fraction up to 15.4 vol % were achieved. With increasing titanium concentration in the molten pool, fractions of TiC and retained austenite increase and the shape of TiC particles changes from cubic to dendritic form. At the same time, the cementite fraction decreases, lowering the overall hardness of the TRL. A good agreement between experimental and calculated results was achieved. Comparative dry sliding wear tests between the as-received DCI, the TRLs and also laser surface melted layers (SMLs) have been performed following the ASTM G 99 standard test method under contact pressures of 2.12 and 4.25 MPa. For both the as-received DCI and the SMLs, the wear rates increased with increasing contact pressure. The TRLs exhibited a significantly higher wear resistance than the others, which was found to be load independent.

## 1. Introduction

Recently, there has been growing interest in the application of surface modification methods to increase the wear resistance of the ductile cast iron (DCI) [1,2]. Considerable research has been devoted to laser surface modification methods, such as laser surface melting (LSM) and alloying (LSA) processes [3,4]. The LSA currently appears to be a leading method due to mainly altering the surface chemical composition [5,6]. Inherent rapid solidification and cooling involved in laser surface modification processes provide a unique opportunity for non-equilibrium synthesis of novel materials [7]. Additionally, the ability to change and accurately control of the chemical composition in the molten pool during LSA, allows the surface properties of the fabricated surface layers to be tailored to the surface requirement of the application [8]. In the case of the DCI, an especially promising approach to improve its wear properties is the in-situ formation of ceramic-reinforced composite surface layers, by introducing elements of a strong carbide forming tendency, such as titanium, into the molten pool [9]. The main advantage of in-situ composite materials as compared to those produced by ex-situ methods is a clean and unoxidized reinforcement-matrix interface, providing more compatible and stronger interfacial bonding [10,11]. Additionally, the reinforcing particles formed during in-situ processing are more thermodynamically stable in the metal matrix [12]. As a result, the in-situ composite materials exhibit superior physical and mechanical properties, such as thermal stability, fatigue strength and wear resistance, making them suitable for application in the power generation, automotive and aerospace industries [13]. Due to very good tribological properties of TiC, composite surface layers reinforced by in-situ synthesized TiC particles have a high potential for application in machine parts working under severe sliding wear conditions [14]. Consequently, several studies have been directly focused on a fabrication of such composite layers via laser surface treatment processes. Liu et al. [15] produced TiC-reinforced surface layers on a grey cast iron substrate using LSA with a pre-placed titanium powder. The resulting composite layers had a significantly higher wear resistance to that of the substrate material. Wang et al. [16] used laser cladding to synthesize in-situ TiC-reinforced Fe-based composite coatings and reported that the coatings exhibited an excellent sliding wear resistance and low frictional coefficient. Emamian et al. [17] have demonstrated composite coatings manufactured via the laser cladding, containing up to about 60 vol % of in-situ formed TiC particles in Fe matrix. They stated that the abrasive wear resistance of the coatings increased with increasing TiC fraction. Although some progress has been made in this field, there are still many unresolved issues regarding the ability to tailor the overall surface layer properties by controlling the size, shape and distribution of the reinforcing phase.

Some published works concerning the in-situ synthesis of composite materials suggest that the experimental results should be complimented by the modelling and simulation of the solidification behaviour of the fabricated alloy system [18,19]. The use of computational thermodynamics allows an evaluation of phase stability in multicomponent systems as a function of temperature and composition and therefore provides the critical basis for understanding phase stability during the in-situ processing of composite materials [20,21]. Moreover, such calculations can be a very useful tool in the optimization of processing conditions and composition of in-situ composite layers [22].

Taking the above into consideration, the present work includes an experimental approach to the in-situ synthesis of TiC phase during the LSA of the DCI substrate coupled with the thermodynamic calculations. In this work, the diode laser surface alloying system with a direct injection of a titanium powder into the molten pool was utilized to the fabrication of TiC-reinforced surface alloyed layers (TRLs) on the ferritic-pearlitic DCI. The main efforts have been directed toward understanding the phase selection during solidification of the TRLs with different titanium content and microstructural evolution in general. The friction and wear properties of TRLs were also evaluated.

## 2. Materials and Methods

Ductile cast iron grade EN-GJS-700-2 was selected as the substrate material. The chemical composition of the used DCI is presented in Table 1. The microstructure of the DCI used consisted of graphite spheres with an average diameter of approx. 30 μm in a pearlitic/ferritic matrix. Rectangular plates of the substrate material 80 mm long, 60 mm wide and 10 mm thick were ground to a surface finish of 0.5 µm R_a_ and cleaned with acetone prior to the LSA process. The alloying material was a commercially available titanium powder (AMPERIT 154, H.C. Starck GmbH, Munich, Germany) having 99% purity and a particle size range of 40–70 μm.

The laser source employed in the present work was a 2 kW continuous wave high power direct diode laser (HPDDL, Rofin-Sinar Laser GmbH, Hamburg, Germany) having a rectangular beam with the top-hat intensity distribution in the slow-axis direction and a near Gaussian in the fast-axis direction. The rectangular laser beam spot of size 1.5 × 6.6 mm was focused at the surface of substrate material and the short axis of the beam was set parallel to the traverse direction. The titanium powder was blown into the molten pool via an off-axis powder delivery system. The used powder delivery system ensures a uniform powder distribution on the surface of the molten pool and a feed rate accuracy of ±0.5%. Details of the used powder delivery system are available elsewhere [23]. Argon was used as a shielding gas.

In order to establish the alloying conditions which would provide the TRLs with the highest possible volume fraction of the TiC particles (for the carbon content in the used DCI substrate) and their homogenous distribution throughout the layer, a series of alloying trials were performed using a laser power range of 1000–2000 W and a traverse speed range of 1.25–3.33 mm/s. Under these processing conditions, the heat input (defined by the ratio of the laser power and the traverse speed) ranged from 300 to 1200 J/mm. The powder feed rate was defined as the amount of titanium powder provided per unit length of the single-pass alloyed bead and its range was 2.0 to 11.0 mg/mm. The first stage of the experimental approach, comprising a fabrication of single-pass alloyed beads, was to determine the impact of main processing parameters on the morphological evolution of the TiC phase and the homogeneity of its distribution throughout the cross-section of the fusion zone, Table 2. The next stage of experimental approach was focused on understanding of the effect of solidification conditions and titanium concentrations in the molten pool on the general microstructure of the TRLs produced via multi-pass overlapping alloying process, Table 3.

To provide a comprehensive analysis of the microstructure evolution of the TRLs, the surface melted layers (SMLs) produced on the used DCI under selected conditions was also considered. Additionally, to achieve a deeper understanding of the microstructure development of TRLs (primary solidifications phases and their stability under rapid- and slow cooling conditions), the analysis of experimental results was preceded by computational thermodynamics. The thermodynamics calculations on the Fe-C-Si-Ti quaternary alloy system were performed under both equilibrium and non-equilibrium conditions using the commercial Thermoc-Calc software. Although other alloying elements are present in the used DCI (in small amounts), it is assumed that their effect on the solidification path is negligible. The Scheil module was used to predict the non-equilibrium solidification paths of the alloy system. This module is based on the Scheil-Gulliver solidification model, which assumes infinitely rapid diffusion in the liquid phase, no diffusion in the solid phase, local equilibrium at the solid/liquid interface and no γ-Fe(FCC) to α-Fe(BCC) transformations.

Microstructural characterization was performed by a scanning electron microscope (SEM, Phenom-World, Eindhoven, The Netherlands and Carl Zeiss Microscopy GmbH, Jena, Germany) equipped with an energy dispersive spectrometer (EDS, EDAX Inc., Mahwah, NJ, USA) and X-ray diffraction (XRD). XRD patterns were obtained using a PANalytical X’Pert PRO MPD X-ray diffractometer equipped with an X’Celerator detector (Malvern Panalytical, Almelo, The Netherlands) and a Co-K*_α_* (λ = 0.179 nm) source. The X-ray tube was operated at 40 kV and 30 mA. Specimens were scanned through 2θ angles ranging from 30° to 125°. The fraction of martensite and retained austenite in the TRLs and SML was evaluated by the Rietveld refinement method using the FullProf software. Area (volume) fractions of TiC and cementite were calculated from SEM micrographs by means of the Nikon digital image processing software (NIS-Elements Basic Research, Nikon Instech Co., Ltd., Tokyo, Japan). Geometrical parameters of the single processed bead were measured using an optical microscope and image analyser software (Eclipse MA100, Nikon Instech Co., Ltd., Tokyo, Japan).

Microhardness tests were performed on cross-sections of TRLs and SMLs using a Wilson Wolpert 401 MVD Vickers (Wilson Wolpert Instruments, Aachen, Germany). Microhardness measurements were made using a 200 g load and a dwell lime of 10 s.

The hardness of the synthesized TiC particles was obtained with a CSM-Instruments Micro/Nano-Combi-Tester equipped with a Berkovich diamond tip (CSM Instruments, Needham, MA, USA). The indentations were carried out at a maximum load of 10 mN and a loading rate of 0.33 mN/s with a hold period of 10s at maximum load. The hardness of TiC particles was calculated according to the Oliver and Pharr method as an average value obtained in 5 tests.

Friction and wear tests of the TRLs were conducted on a pin-on-disk apparatus designed in accordance with standard ASTM G 99-95a (tribometer T-01M manufactured at the Institute for Sustainable Technologies—National Research Institute, Radom, Poland). Wear discs (40 mm in diameter) after LSA contained single processed bead of 28 mm dimeter. The surface of the discs was ground to R_a_ = 0.1 µm and ultrasonically cleaned in acetone before tests. The pins were made of AISI 52100 steel and were of 3 mm diameter and 800 HV10 hardness. The tests were conducted at contact pressures of 2.12 and 4.25 MPa. The frictional force was measured continuously using a load cell having a sensitivity of 0.1 N. The sliding velocity and distance were 0.6 m/s and 6000 m, respectively. All wear tests were performed under dry conditions in room temperature air and relative humidity of 50%. Each test ran was repeated twice to verify the reproducibility of the data. To determine the degree of wear quantitatively, depth profiles of the wear tracks were measured using a Talysurf profilometer (Taylor Hobson, Leicester, UK). The volume loss was estimated from the average area of the wear profile (measured at four positions perpendicularly to the wear track) and the diameter of the wear tracks. The wear rates (mm^3^/m) were calculated by dividing the volume loss by the total sliding distance. Scanning electron microscopy, in the secondary electron (SE) and backscattered electron (BSE) modes were employed to assess the mechanism of material removal.

## 3. Results and Discussion

### 3.1. Thermodynamic Calculations

**Figure 1:**
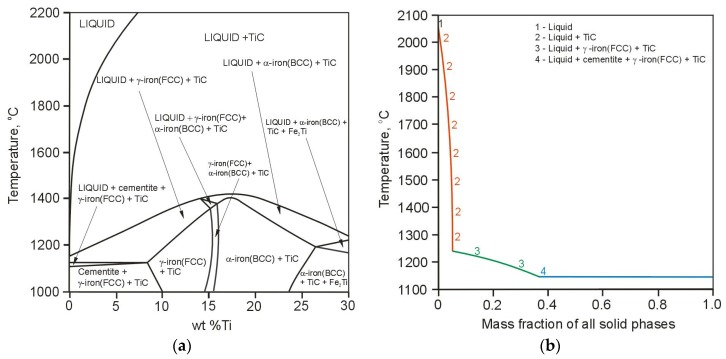
(**a**) Calculated phase diagram for the quaternary Fe-3.6 wt % C-2.5 wt % Si-Ti alloy system; (**b**) Scheil solidification path of the Fe-3.6 wt % C-2.5 wt % Si-4 wt % Ti alloy system.

**Figure 2:**
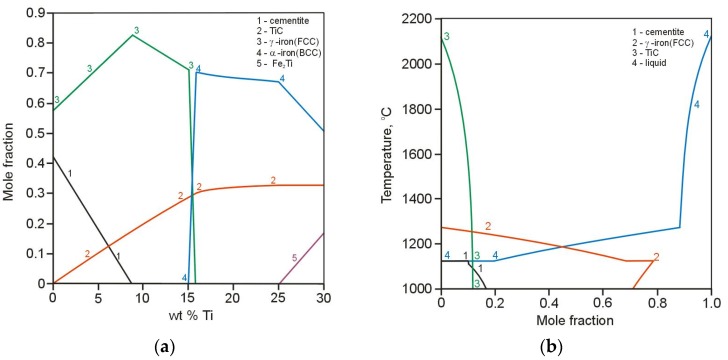
(**a**) Effect of the amount of titanium in the Fe-3.6 wt %C-2.5 wt % Si-Ti alloy system on the mole fraction of all phases calculated at 1100 °C; (**b**) thermal stability of the phases in the Fe-3.6 wt % C-2.5 wt % Si-6.0 wt % Ti alloy system.

Figure 1a presents the equilibrium phase diagram resulting from the computational calculations of the quaternary Fe-3.6 wt % C-2.5 wt % Si-Ti alloy system of the TRLs. The solidification path of the above alloy system containing 4.0 wt % of Ti, calculated based on Scheil-Gulliver model, is presented in Figure 1b. Additionally, the effect of titanium content in the molten pool on the number of precipitated phases is shown in Figure 2a. Based on the Scheil prediction, which indicated that the terminal solidification temperature was approx. 1150 °C, the diagram shown in Figure 2a was generated at 1100 °C for 1 mole system. The Scheil model showed that the first phase to precipitate in the molten pool is TiC phase. On further cooling, the formation of primary austenite γ-Fe(FCC) dendrites occurs. In the last stages of solidification process, the precipitation of cementite was predicted. According to the calculations, the amount of TiC in the TRL increases gradually, as the content of titanium increases, until approx. 16 wt % Ti and then remains constant at a level of 0.31 mole fraction. This amount of TiC is the result of a complete reaction between titanium and carbon in the molten pool and is generally limited by the carbon content in the substrate material. Note that the composition of TiC phase predicted by the calculations was stochiometric TiC_1.0_. Assuming that during the investigated alloying process (using different powder feed rates), the carbon content in the molten pool remains constant, the maximum amount of TiC particles which can be achieved is about 20 wt %. The calculations showed that the cementite phase is formed up to approx. 9 wt % Ti. A titanium content slightly higher than about 9 wt %, the microstructure of the TRLs should contain TiC particles in the matrix consisted of martensite and also some amount of retained austenite, as a result of high cooling rates associated with the laser processing. The literature concerning the laser treatment of the cast irons suggests that under non-equilibrium cooling conditions, the austenite becomes supersaturated in carbon, thus suppressing the martensitic transformation [24,25]. Based on the thermal analysis of the investigated laser alloying process, which will be presented in the separate paper, it was found that the cooling rates in the optimal range of processing parameters are of the order of 10^3^ K/s. The presence of austenite in both TRLs and also SML was confirmed by XRD analysis. Taking into account that the martensitic matrix provides better support for reinforcing particles than austenitic in the composite layers under the wear conditions [26], the amount of retained austenite should be restricted. It is important to remark that the carbon supersaturation of austenite would be limited by increasing titanium content in the molten pool, due to the precipitation of TiC phase. This claim is consistent with the calculations indicating that the TiC presents significantly higher thermal stability compared to the austenite. The calculations of the thermal stability of the phases, performed in the range of 2200 °C to 1000 °C with 1 mole of the Fe-3.6 wt % C-2.5 wt % Si-6.0 wt % Ti alloy system, indicated that at this titanium content the TiC is stable up to 2100 °C, whereas the austenite disappears at about 1280 °C, Figure 2b. Moreover, Figure 1a shows that the thermal stability of TiC increases with increasing Ti content. As can be seen in Figure 1a and Figure 2a, the formation of the intermetallic compound Fe_2_Ti was predicted for titanium contents higher than approx. 25 wt %.

### 3.2. Macro and Micro Analysis

**Figure 3:**
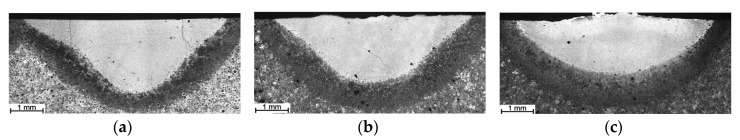
Micrographs of (**a**) single-pass melted bead no. M1 (Table 2) and single-pass alloyed beads no. (**b**) A1; (**c**) A3.

**Figure 4:**
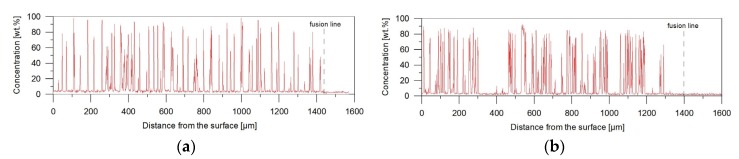
Titanium concentration profiles on the cross-section of single-pass alloyed beads no. (Table 2): (**a**) A3; (**b**) A4. Depth profiles in the centre of the bead.

Figure 3 compares the cross-section of the single-pass melted bead with cross-sections of single-pass alloyed beads produced at the same heat input and different powder feed rates (Table 2). It is well known that the mass transport in the molten pool and thereby the resulting concentration profile of the alloying material in the laser alloyed bead is directly influenced by the intensity and pattern of the fluid flow (Marangoni convection) in the molten pool [27]. The shape of these fusion zones suggests that during both the LSM and LSA processes the surface tension temperature coefficient on the molten pool surface was positive, i.e., the surface tension was highest at the centre of the molten pool and produced fluid flow inward along the surface of the molten pool. In general, this pattern of the fluid flow results in a formation of the hemispherical molten pool. As the laser power increases, the convection becomes much faster, leading to the narrower and deeper molten pool [28]. As can be seen in Figure 3a, during the LSM process the above-mentioned heat and mass transfer mechanism was dominant. However, in the case of the LSA process, it can be concluded that the addition of titanium into the molten pool led to the decrease in the intensity of the convection. This suggestion is supported by measurements of geometrical parameters of the fusion zone, summarised in Table 2. With the increase of the titanium concentration in the molten pool, under a constant laser power and a traverse speed, the depth of the fusion zone decreased and the bead profile became more hemispherical in shape. Simultaneously, the cross-sectional area of the bead was markedly reduced. It should be emphasized that the TiC particles are the result of an exothermic reaction between liquid titanium and carbon in the molten pool. Consequently, the higher fraction of TiC particles, associated with the higher titanium content, indicates a larger amount of the heat generated directly in the molten pool, what should bring about the larger fusion zone area. Based on the above, it is reasonable to conclude that the convective heat transfer in the molten pool becomes gradually reduced with the increasing TiC fraction. The resultant reduction in the mass transport in the molten pool (throughout the fusion zone) is reflected on Ti concentration profiles on the cross-section of single-pass alloyed beads, presented in Figure 4. These results are in agreement with those of Yuan et al. [29]. They formed TiC-reinforced layers on a Ti6Al4V substrate using LSA and stated that the convection in the molten pool is the main factor controlling the fraction of TiC particles and their distribution throughout the alloyed region.

**Figure 5:**
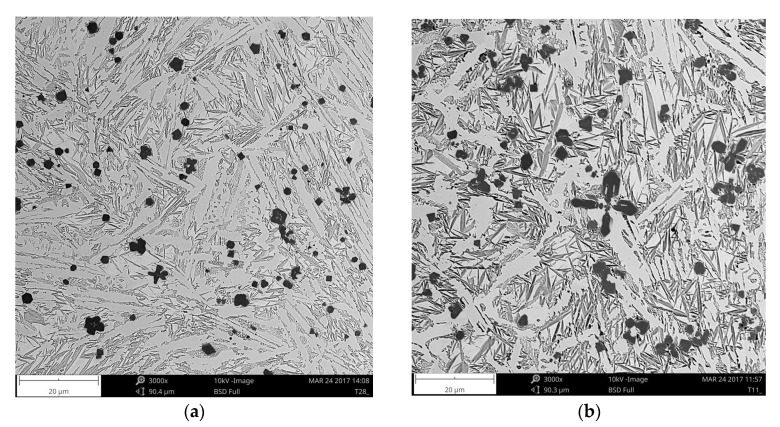

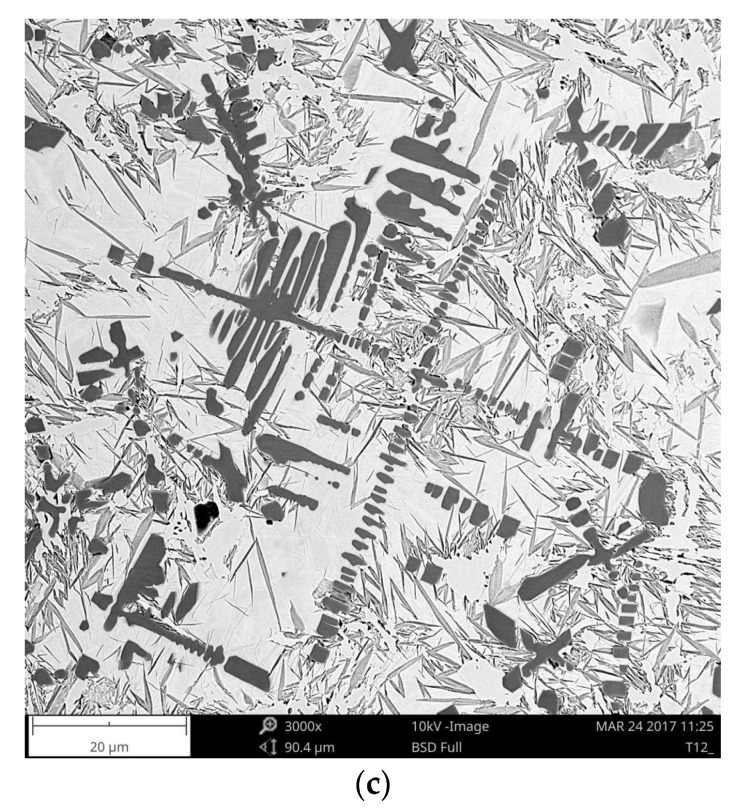
SEM micrographs taken from the mid-section of single-pass alloyed beads (Table 2): (**a**) A1; (**b**) A2; (**c**) A3.

**Figure 6:**
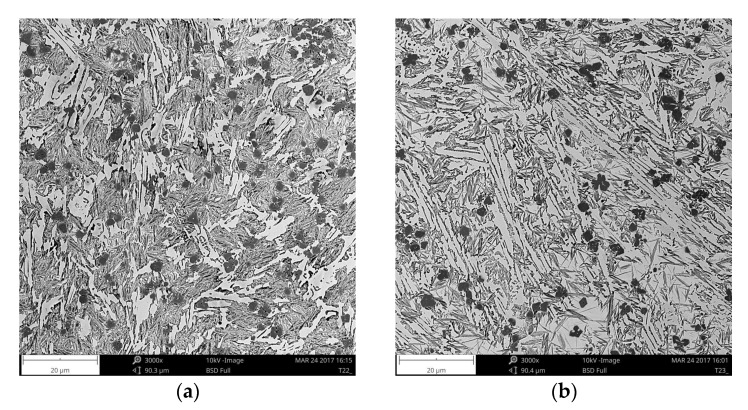
SEM micrographs taken from the mid-section of single-pass alloyed beads no. (Table 2): (**a**) A5; (**b**) A6.

**Figure 7:**
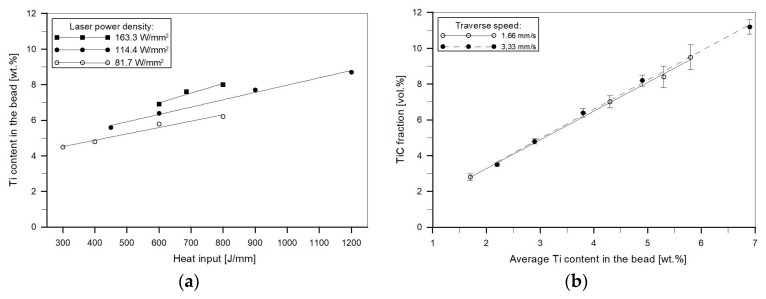
(**a**) Effect of the heat input level and laser power density on the maximum titanium content in the uniformly alloyed bead; (**b**) effect of the titanium concentration in the molten pool and traverse speed (at constant heat input of 600 J/mm) on the TiC volume fraction.

**Figure 8:**
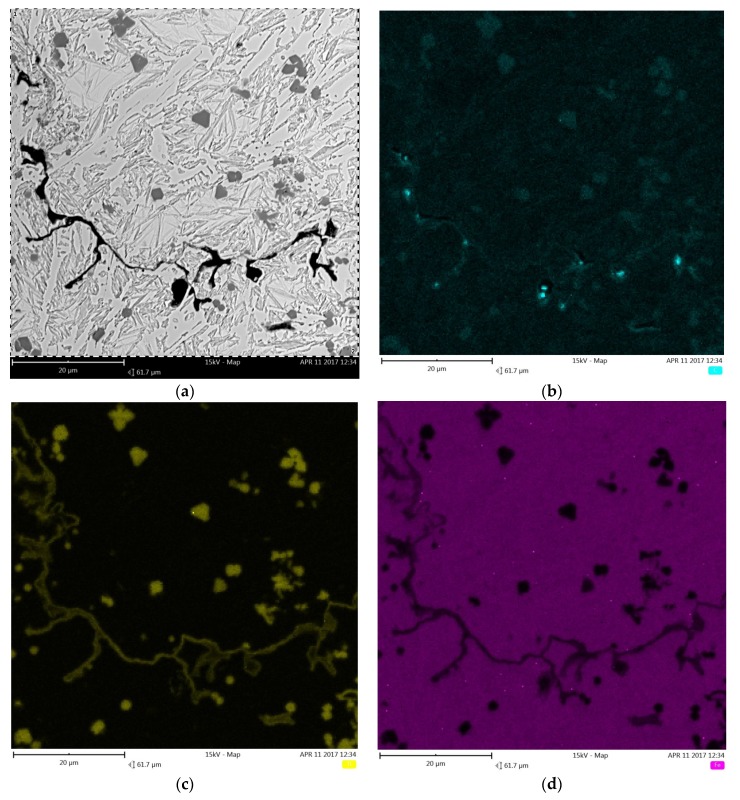
(**a**) SEM micrograph taken from the mid-section of the single-pass alloyed bead with an excessive titanium content showing a morphology of micro voids; (**b**–**d**) corresponding maps of C, Ti and Fe distribution, respectively.

Cross-sectional SEM micrographs taken from the mid-section of single-pass alloyed beads with different titanium contents produced at the heat input of 1200 J/mm are presented in Figure 5. The microstructure of the beads contains TiC particles, primary austenite dendrites partially transformed to martensite and a ledeburite structure in the interdendritic regions. The TiC particles were observed in both the dendrites and interdendritic regions. This is consistent with the thermodynamic calculations that predicted a significantly higher thermal stability of the TiC phase than that of the austenite phase. The metallographic data, presented in Table 2 and the micrographs, depicted in Figure 5 and Figure 6, clearly show that the volume fraction of TiC particles and their size are directly influenced by the titanium concentration in the molten pool. Qualitative analysis of micrographs indicated that at the heat input of 1200 J/mm, TiC fractions in the beads with the average titanium content of 3.0 and 8.7 wt % were 4.8 ± 0.31 and 14.8 ± 1.45 vol %, respectively. The morphology of TiC particles changes at the same time from mainly cubic with particles size < 6 µm to dendritic with size up to 50 µm. A similar growth of the TiC phase in the molten pool during laser surface modification processes has been reported in several papers [17,30]. It should be pointed out that the change in the traverse speed, in the investigated range of 1.25–3.33 mm/s, did not influence either the TiC fraction or their size (Figure 6 and Figure 7b). It indicates that the resulting solidification conditions allowed the complete reaction between carbon and the entire amount of titanium introduced into the molten pool. This finding is supported by a minor titanium content in the martensitic-austenitic matrix. From the analysis of micrographs, it is also clear that the processing variables that directly determine the maximum titanium content in the uniformly alloyed bead are both the heat input level and the power density. As can be seen in Figure 7a, there is a general trend towards the maximum titanium content increase along with the increasing heat input. However, it should be noted that the increase in the power density, at the constant heat input level (by adjusting traverse speed), leads to further increase in the maximum titanium content. This is due to the fact that the higher power density gives rise to more intensive fluid flow in the molten pool and as a result more efficient transfer of alloying material throughout the fusion zone. In the investigated processing parameters, the maximum titanium content was found to be about 8.7 wt %. At this titanium content, the TiC fraction in the bead was approx. 14.8 vol %. An excessive powder feeding rate for the given heat input level and the power density led to a non-uniform composition (Figure 4b), porosity and formation of micro-voids. A morphology of the formed micro-voids is presented in Figure 8a. The corresponding EDS compositional maps shown in Figure 8b–d revealed that the micro-voids are a consequence of an incomplete dissolution of graphite nodules. It is important to remark that a cracking tendency of single-pass alloyed beads was gradually decreased with the increasing titanium content. In contrast, the single-pass melted beads are highly prone to a formation of a crack network.

**Figure 9:**
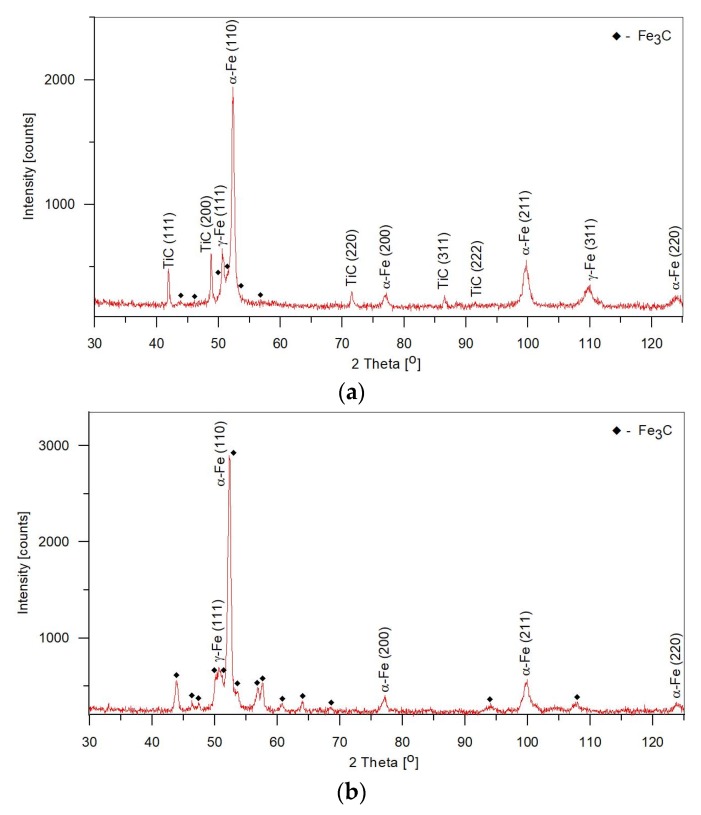
XRD patterns of (**a**) the TRL no. 3 (Table 3) and (**b**) the SML produced under processing condition no. M1 (Table 2) and overlap ratio of 30%.

Microstructural parameters of the TRLs produced under different processing conditions, based on a quantitative analysis of micrographs and XRD patterns, are presented in Table 3. Representative XRD patterns for the TRL no. 3, containing the highest TiC fraction (~15.4 vol %) and the SML produced at the same heat input are depicted in Figure 9a,b, respectively. Fractions of cementite and retained austenite in the above SML were measured to be 53 ± 2.5 vol % and 3.9 ± 0.8 wt %, respectively. As the thermodynamic calculations predicted, in the case of the TRLs, with increasing titanium content, at the same heat input and traverse speed, the fraction of cementite gradually decreased as a consequence of the increasing fraction of TiC phase. However, the data presented in Table 3 imply that simultaneously the fraction of the retained austenite was raised. Moreover, the increase in the traverse speed, at the constant heat input level (by adjusting laser power) and powder feed rate, results in a marked increase in the amount of retained austenite. At the heat input of 600 J/mm, the amount of retained austenite in the TRLs having the same titanium content of about 7.5 wt % was measured to be 20.7 ± 0.9 wt % and 30.9 ± 1.2 wt % for the traverse speed of 1.66 and 3.33 mm/s, respectively. At the same time, fractions of the TiC and the cementite, for both TRLs, remained unchanged at about 14 and 19 vol %, respectively. The variation in the fraction of retained austenite can be attributed to a change in carbon content in the primary austenite grains caused by different cooling rates. Faster cooling rates increase the carbon content in the austenite, what suppresses the martensitic transformation [31]. Therefore, lower traverse speeds and the resulting lower solidification and cooling rates, are favourable for providing higher fractions of the martensite in the matrix of the composite layers presently being considered. This, in turn, will provide better support of the TiC reinforcing particles during sliding wear.

The lattice parameter of TiC phase (calculated from the XRD patterns) was evaluated to be 0.433 nm. Moreover, it has been noted that the lattice parameter did not change in the whole range of processing conditions. It is well established that the lattice parameter of TiC phase depends on the carbon content [32]. Based on data reported by Holt and Munir [33], it can be concluded that the reaction between titanium and carbon taking place in the molten pool, under investigated solidification conditions, led to the formation of a stochiometric TiC_1.0_ phase. This composition has been confirmed by nanoindentation testing. It is well known that the hardness of the TiC phase varies with the carbon content, i.e., the hardness decreases with a decrease in carbon content [34]. The average hardness and elastic modulus of the TiC particles were found to be 45 ± 0.8 GPa and 461 ± 19 GPa, respectively. These values are in good agreement with these reported in the literature for the stochiometric TiC carbide [35].

### 3.3. Hardness Analysis

**Figure 10:**
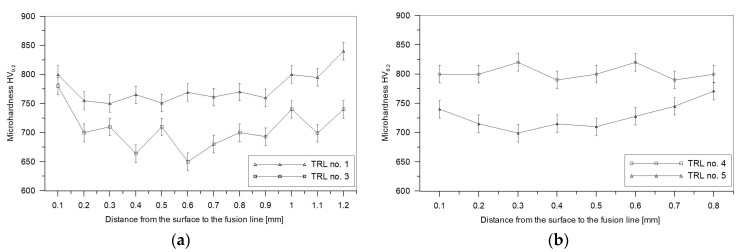
Microhardness profiles for the TRLs (Table 3): (**a**) no. 1 and 3; (**b**) no. 4 and 5.

Microhardness profiles of the TRLs produced at different processing conditions are depicted in Figure 10. In general, the TRLs exhibited homogeneous hardness distribution across the layer, which confirms their compositional homogeneity. The microhardness data show a general trend toward decreasing the total hardness of the TRL with an increase in titanium content for the given heat input and traverse speed (Figure 10a). Moreover, as can be seen in Figure 10b, at the given heat input and titanium content, there is a trend for the TRL’s hardness to decrease with the increasing traverse speed. The above variations in hardness are attributed mainly to changes in fractions of cementite and retained austenite. In the former case, the hardness reduction is directly associated with a decrease in the cementite fraction. While, in the latter case, the lower hardness results directly from a significantly higher amount of the retained austenite. Note that the average hardness value for the SML, that is the layer containing the highest fraction of cementite (~53 vol %), was 850 ± 40 HV. Compared with the SML, the TRLs having the cementite fractions of about 8.1 (TRL no. 3) and 20.1 (TRL no. 4) vol % exhibited an average hardness of 703 ± 35 and 802 ± 11 HV, respectively. Note that these TRLs contain almost the same fractions of TiC and retained austenite. The major contribution of the cementite fraction in the overall hardness of the TRLs developed on a cast iron substrate has already been reported by Park et al. [36]. As pointed out in the section on microstructure, the decrease in the fraction of cementite is related to a simultaneous increase in the fraction of TiC phase. It means that in the investigated alloy system the fraction of TiC has no significant effect on the hardness.

### 3.4. Sliding Wear Behaviour

**Figure 11:**
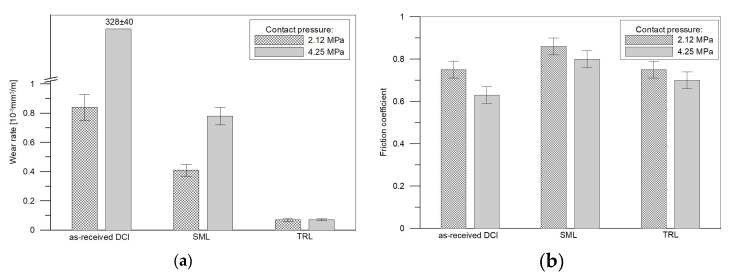
Comparisons of (**a**) wear rates and (**b**) the friction coefficient for the as-received DCI, SML and TRL tested at different normal pressures. The error bar indicates the variations from the mean value due to duplicated tests.

Based on the metallographic data, the TRL no. 3 (Table 3) containing the highest TiC fraction, was selected for the dry sliding wear test. The wear testing of specimens of the DCI in the as-received condition and also after laser surface melting (the SML produced under processing condition no. M1, Table 2) was conducted for comparative purposes. Figure 11a shows the wear rate comparison of the as-received DCI, SML and the TRL at contact pressures of 2.12 and 4.25 MPa. The corresponding values of the friction coefficient are presented in Figure 11b. The data clearly imply that the as-received DCI showed the highest wear rates under the applied loads. Additionally, the friction behaviour of the as-received DCI was a strong function of the contact pressure. The LSM process significantly improved the wear resistance of the DCI, approx. 2 and 420 times at contact pressures of 2.12 and 4.25 MPa, respectively. The formation of the TRLs provided further improvement of wear properties of the used DCI substrate. The TRL containing ~15 vol % of TiC exhibited about 6 and 11 times lower wear rates that the SML for contact pressures of 2.12 and 4.25 MPa, respectively. Another remarkable feature of the relationship evident in Figure 11a is that the TRL at both applied loads showed a marginal difference in wear rate, while the wear rates for the SMLs and especially for the as-received DCI were highly load-dependent. Regardless of the type of the tested material, the coefficient of friction decreased with the contact pressure. The SMLs revealed the highest friction coefficient values of 0.86 and 0.80 at 2.12 MPa and 4.25 MPa, respectively. At 2.12 MPa, the coefficient of friction of the as-received DCI and the TRLs was, in general, the same approx. 0.75. As the pressure increased to 4.25 MPa, the as-received DCI showed the lowest friction coefficient about 0.63, while the TRL demonstrated the friction coefficient of 0.70.

**Figure 12:**
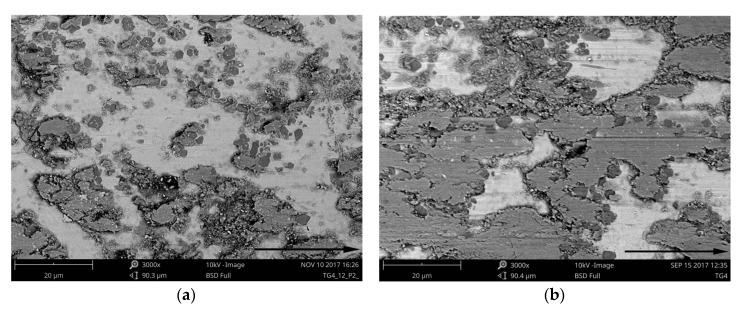
BSE SEM images of the TRL after wear at contact pressures of: (**a**) 2.12 MPa; (**b**) 4.25 MPa. Arrows indicate sliding direction.

**Figure 13:**
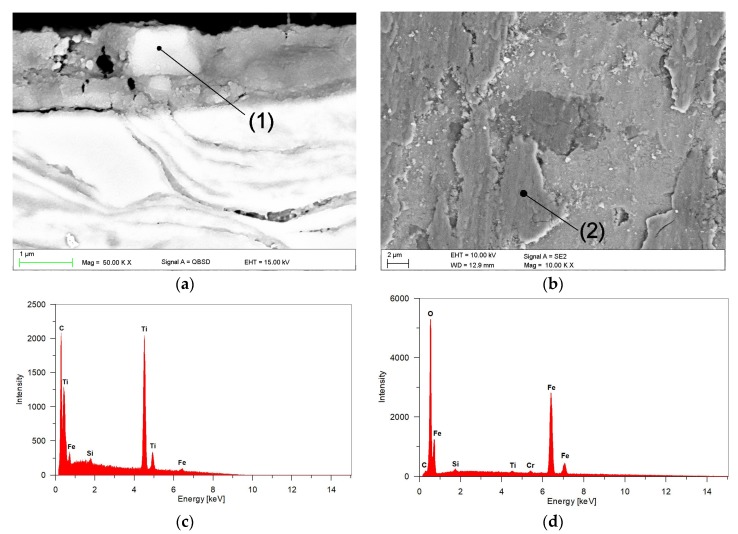
(**a**) BSE SEM image of the subsurface region of TRL after a sliding wear at 4.25 MPa for 6000 m showing the cross-sectional morphology of the tribo-layer; (**b**) corresponding SE SEM image of the worn surface; (**c**,**d**) EDS spectra from regions (1) and (2), respectively, in (**a**,**b**).

**Figure 14:**
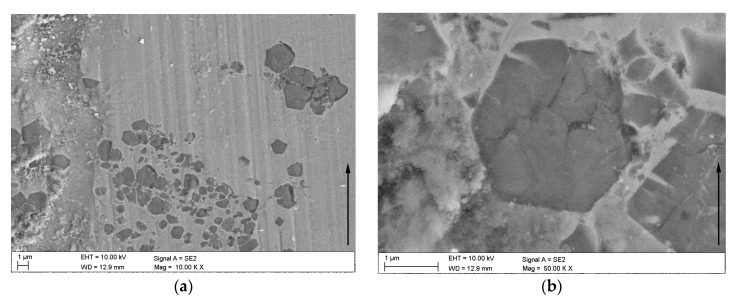
SE SEM images of (**a**) the worn surface of the TRL tested at 2.12 MPa; (**b**) a detail from (**a**) showing cracking of TiC particles. Arrows indicate sliding direction.

The morphology of the worn surfaces of the TRL is presented in Figure 12, Figure 13 and Figure 14. As can be seen, the presence of the tribo-layer, resulting from a material transfer from the counterface to the surface of the TRL, is evident at the two applied contact pressures. Backscattered electron images in Figure 12, where brighter contrast corresponds to a higher average atomic number, clearly indicate that the amount of the transferred material remarkably increased with the contact pressure. As a result, at higher contact pressure, the tribo-layer covers a significant portion of the wear track, creating a third-body contact. 

**Figure 15:**
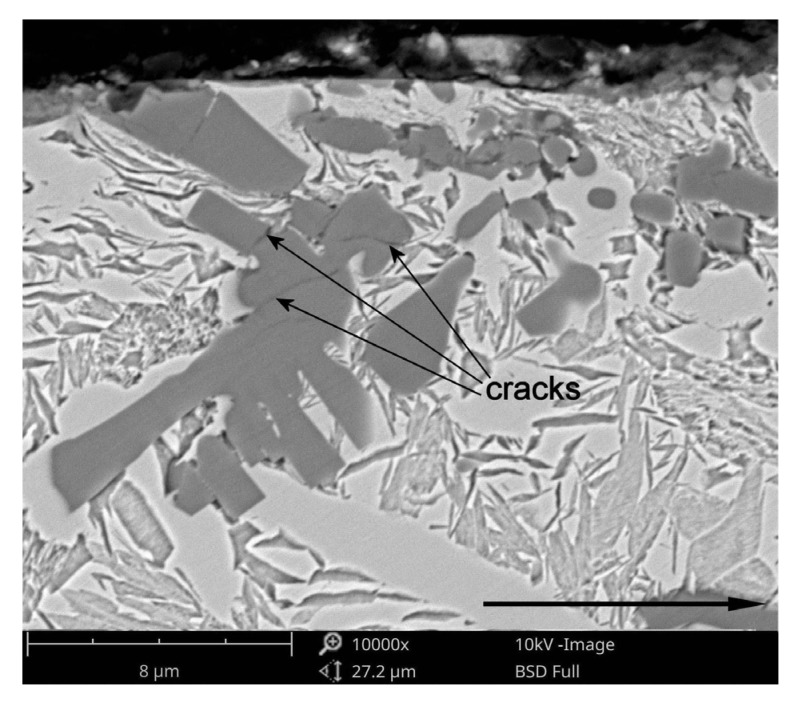
BSE SEM image of the subsurface region of the TRL after a sliding wear at a contact pressure of 4.25 MPa for 6000 m showing cracking of the TIC phase. Arrow indicates sliding direction.

**Figure 16:**
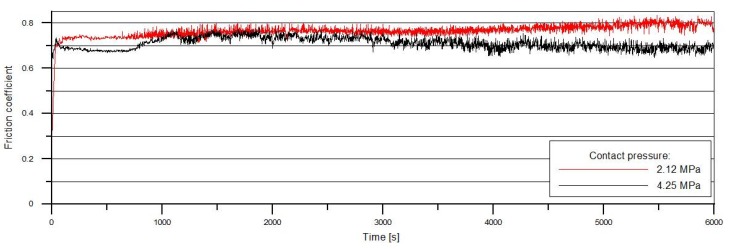
Friction coefficient versus time data for the TRL under different contact pressures.

EDS analysis of the tribo-layer, Figure 13d, indicated the presence of Fe, Ti, Cr and O. Moreover, the backscattered electron image of the cross-section of the worn surface at 4.25 MPa, Figure 13a, revealed a number of fine TiC particles embedded in the tribo-layer. The presence of broken TiC particles in the tribo-layer, confirmed by EDS analysis presented in Figure 13c, is consistent with the fact that the TiC phase is highly brittle. This results in severe cracking of this phase even at a lower contact pressure. Higher magnification of the worn surface at 2.12 MPa, as shown in Figure 14b, showed intensive cracking and chipping of the TiC particles. The brittle nature of the TiC phase is clearly seen in Figure 15, which gives details of the subsurface features of the worn TRL specimen. As can be observed, the subsurface cracks in the TiC phase, that run mainly parallel to the worn surface, are also present at a distance of 8 µm beneath the worn surface. The above prove that the tribo-layer formed under a contact pressure of 4.25 MPa is, in general, a mixture of a partially oxidized pin material and fine TiC particles. The presence of the tribo-layer, during sliding at a contact pressure of 4.25 MPa, limited the extent of direct contact between the matrix material of the TRL and the hard asperities of the mating pin surface, resulting in decreasing wear intensity. Some researchers have reported the formation of such mechanically mixed layer during the dry sliding wear of MMCs [37]. AlMangour et al. [13] stated that in-situ formed very fine TiC particles (~100 nm in size) uniformly distributed in Fe-based matrix tend to form the tribo-layer over the entire worn surface, significantly increasing wear resistance. No attempt was made to quantify the thickness of the tribo-layer, as the thickness was not uniform across the wear track. However, it can be seen in Figure 13a that the thickness of the layer on the worn surface of TRLs at 4.25 MPa after 6000 m could be as large as 1.5 µm. The formation of the tribo-layer is also reflected in the friction curves, presented in Figure 16. The decrease in the friction coefficient during the steady-state wear stage was believed to be the result of the gradual build-up of the tribo-layer. However, some areas of the wear track, as shown in Figure 12 and Figure 13, showed the evidence of cracking and delamination of this tribolayer.

In contrast to the surface worn at a contact pressure of 4.25 MPa, the worn surface after testing at 2.12 MPa showed a relatively smooth wear track with occasional regions covered by the tribo-layer, Figure 12a. It seems that the tribo-layer formed at the lower contact pressure is much more brittle than that formed at higher pressures. This can be attributed to the higher degree of oxidation of the transferred pin material. As a result, the tribo-layer becomes highly prone to cracking and delamination. The resultant wear debris lead to intensive ploughing and microcutting of the matrix material, Figure 14a. Under these conditions, the wear is controlled primarily by an abrasive-type wear mechanism. The occurrence of such wear mechanism is also confirmed by the increase in the friction force, Figure 16.

## 4. Conclusions

In order to improve wear resistance of the DCI EN-GJS-700-2 grade, TiC-reinforced surface layers (TRLs) have been in-situ formed via LSA with a direct injection of a pure titanium powder into the molten pool. It was found that the fraction of TiC particles in the TRLs increases with the increase of titanium concentration in the molten pool. The shape of the TiC particles can be modified from cubic to dendritic form by increasing the titanium concentration. However, the amount of titanium which can be introduced into the molten pool during fabrication of the uniformly alloyed single-pass bead was found to be approx. 8.7 wt %. This limitation is a consequence of a gradual decrease in the intensity of the fluid flow in the molten pool with increasing TiC fraction. In the range of optimal processing conditions, that is providing a uniform TiC distribution, the morphology and fraction of the TiC particles were not affected by a change in the traverse speed. In general, a good agreement between experimental and calculated results has been achieved. With the increase in the titanium concentration the fraction of cementite decreases, whereas the fraction of retained austenite in the matrix increases. Moreover, at a given titanium content, the fraction of the retained austenite is highly affected by the traverse speed, due to a change in cooling and solidification rates. Fractions of the cementite and retained austenite have a major contribution to the overall hardness of the TRLs.

The dry sliding wear resistance of the TRLs containing approx. 15 vol % of TiC particles was notably higher than those of the as-received DCI and SMLs. The TRLs showed a marginal difference in the wear rate under testing at contact pressures of 2.12 and 4.25 MPa, while the wear rates for the SMLs and especially for the as-received DCI were highly pressure dependent. Low wear rates of the TRLs under higher applied contact pressure were associated with the formation of the mechanically mixed tribo-layer on the worn surface, which acted as an additional wear protection layer. Moreover, it was considered that the tribo-layer is responsible for lowering the friction coefficient of the TRLs under higher contact pressures.

Based on the thermodynamic calculations, it was hypothesized that further increase of titanium concentration in the molten pool, above the level obtained in the present study, would both provide higher TiC fraction and decrease the fraction of retained austenite in the matrix of the TRLs. The evaluation of the hypothesis will be conducted in future experiments and presented in a separate paper. The approach to that study will concern the use of the additional alloying material influencing the intensity of the fluid flow in the molten pool.

## Figures and Tables

**Table 1:** Chemical composition of the used DCI grade EN-GJS-700-2 (wt %).

C	Si	Cu	Mn	Cr	Ni	S	P	Fe
3.60	2.51	0.78	0.25	0.02	0.04	0.008	0.016	balance

**Table 2:** Selected processing parameters of the LSA process and corresponding geometrical parameters of the fusion zone of single-pass alloyed bead, Ti contents and TiC fractions in the bead.

Processing Condition No./Bead No.	Laser Power (W)	Traverse Speed (mm/s)	Heat Input (J/mm)	Powder Feed Rate (mg/mm)	Cross-Sectional Bead Geometry		Ti Content (wt %)	TiC Fraction (vol %)
					Fusion Zone Depth (mm)	Fusion Area of the Bead (mm^2^)		
A1	1500	1.25	1200	2.0	1.94	7.29	3.0 ± 0.20	4.8 ± 0.31
A2	1500	1.25	1200	6.0	1.79	7.09	5.9 ± 0.61	9.4 ± 1.21
A3	1500	1.25	1200	10.0	1.63	6.94	8.7 ± 1.09	14.8 ± 1.45
A4	1500	1.25	1200	11.0	1.54	6.85	N ^2^	-
A5	1000	1.67	600	4.0	1.17	5.29	5.7 ± 0.94	9.3 ± 1.39
A6	2000	3.33	600	4.0	1.34	5.55	5.5 ± 0.45	8.9 ± 0.61
M1 ^1^	1500	1.25	1200	-	2.24	7.59	-	-

^1^ single-pass melted bead produced for comparative purpose; ^2^ non-uniform composition.

**Table 3:** Effect of the processing variables on the microstructural parameters of the TRLs.

TRL no.	Processing Condition no. (Table 2) ^1^	α-Fe (Martensite) Fraction (wt %)	Retained Austenite Fraction (wt %)	Cementite Fraction (vol %)	TiC Fraction (vol %)
1	A1	47.2 ± 1.2	6.2 ± 0.1	42.3 ± 1.9	6.44 ± 0.41
2	A2	48.0 ± 1.9	13.4 ± 1.3	31.3 ± 2.2	11.0 ± 1.16
3	A3	66.4 ± 1.1	15.3 ± 1.4	8.1 ± 2.9	15.4 ± 2.11
4	A5	49.8 ± 1.3	20.7 ± 0.9	20.1 ± 2.5	14.1 ± 1.55
5	A6	41.7 ± 1.6	30.9 ± 1.2	18.2 ± 2.0	13.8 ± 1.03

^1^ overlap ratio: 30%.
